# Cerebellar volume as imaging outcome in progressive multiple sclerosis

**DOI:** 10.1371/journal.pone.0176519

**Published:** 2017-04-24

**Authors:** Matilde Inglese, Maria Petracca, Enricomaria Mormina, Anat Achiron, Rebecca Straus-Farber, Shmuel Miron, Michelle Fabian, Stephen Krieger, Aaron Miller, Fred Lublin, Maria Pia Sormani

**Affiliations:** 1 Department of Neurology, Icahn School of Medicine at Mount Sinai, New York, NY, United States of America; 2 Department of Radiology Icahn School of Medicine at Mount Sinai, New York, NY, United States of America; 3 Department of Neuroscience, Icahn School of Medicine at Mount Sinai, New York, NY, United States of America; 4 Department of Neuroscience, Rehabilitation, Ophthalmology, Genetics, and Mother-Child health, University of Genoa, and IRCCS Azienda Ospedale Università San Martino-IST, Genova, Italy; 5 Department of Biomedical Sciences and of Morphologic and Functional Images, University of Messina, Messina, Italy; 6 Multiple Sclerosis Center, Sheba Medical Center, Tel Hashomer, Israel; 7 Sackler Faculty of Medicine, Tel-Aviv University, Tel-Aviv, Israel; 8 Department of Neurology, Columbia University, New York, NY, United States of America; 9 Biostatistics Unit, University of Genoa, Genoa, Italy; Banner Alzheimer's Institute, UNITED STATES

## Abstract

**Background and purpose:**

To assess whether cerebellar volumes changes could represent a sensitive outcome measure in primary-progressive MS.

**Material and methods:**

Changes in cerebellar volumes over one-year follow-up, estimated in 26 primary-progressive MS patients and 20 controls with Freesurfer longitudinal pipeline, were assessed using Wilcoxon test and tested for their correlation with disability worsening by a logistic regression. Clinical worsening was defined as EDSS score increase or change of >20% for 25-foot walk test or 9-hole peg test scores at follow-up. Sample sizes for given treatment effects and power were calculated. The findings were validated in an independent cohort of 20 primary-progressive MS patients.

**Results:**

Significant changes were detected in brain T1 lesion volume (p<0.01), cerebellar T2 and T1 lesion volume (p<0.01 and p<0.05), cerebellar volume, cerebellar cortex volume, and cerebellar WM volume (p<0.001). Only cerebellar volume and cerebellar cortex volume percentage change were significantly reduced in clinically progressed patients when compared to patients who did not progress (p<0.01; respectively AUC of 0.91 and 0.96). Cerebellar volume percentage changes were consistent in the exploration and validation cohorts (cerebellar volume -1.90±1.11% vs -1.47±2.30%; cerebellar cortex volume -1.68±1.41% vs -1.56±2.23%). Based on our results the numbers of patients required to detect a 30% effect are 81 per arm for cerebellar volume and 162 per arm for cerebellar cortex volume (90% power, type 1 error alpha = 0.05).

**Conclusions:**

Our results suggest a role for cerebellar cortex volume and cerebellar volume as potential short-term imaging metrics to monitor treatment effect in primary-progressive MS clinical trials.

## Introduction

One of the barriers to assessing potential neuroprotective agents in multiple sclerosis (MS) is the slow rate of disability accrual. Unfortunately, clinical outcome measures exhibit poor sensitivity when applied to small groups of patients over relatively short periods of time. MRI surrogates are more sensitive to disease activity and current MRI measures such as number of Gadolinium-enhancing and new T2 lesions are useful to monitor response to antinflammatory agents in patients with relapsing-remitting MS. However, they are quite insensitive to changes in patients with primary-progressive MS (PP-MS) where the underlying pathology is dominated by diffuse brain GM and WM damage, and worsening of tissue damage within existing lesions rather than by accumulation of new brain WM lesions[1].

While both brain and cerebellar cortex are major predilection sites for demyelination in patients with primary and secondary progressive MS[2–8], the clinical impact of cerebellar volume over short-term disease progression in PP-MS has received less attention[9,10]. Due to its multiple connections to the forebrain, the thalamus, and the spinal cord, the cerebellum is not only affected by focal WM and GM lesions but also by the secondary degeneration of multiple afferent and efferent connections to the supratentorial brain areas and to the spinal cord. Hence, we hypothesize that cerebellar neurodegeneration might occur at a significant rate with higher chances to impact the patients’ clinical outcome due to the cerebellar strategic position in the motor, coordination and cognitive networks.

The aims of this study were: a) to measure global and cortical cerebellar volume changes in PP-MS patients over one-year follow-up; b) to assess whether changes in cerebellar volume correlated with short-term clinical progression and, c) to determine sample sizes required to demonstrate reduction of cerebellar volume as an outcome measure in a placebo-controlled trial for PP-MS.

## Methods

### Subjects

Twenty-six patients who met the modified McDonald diagnostic criteria[11] and presented a primary-progressive (PP) course[12] were prospectively enrolled. Twenty sex- and age-matched healthy subjects served as controls (CTRLs) (11F/9M; mean age, 51.1 years; range, 34–63 years) for the comparison of MRI metrics. Inclusion criteria for PP-MS patients were: (i) age between 25–65 years; (ii) an Expanded Disability Status Scale (EDSS)[13] lower than 6.5 at screening visit; (iii) disease duration up to 15 years. The use of immuno-modulatory drugs was allowed but, if treated, patients had to be on current treatment for at least one-year. At screening visit, 12 patients were under immuno-modulatory treatment with either glatiramer acetate, interferon β-1a or fingolimod. Exclusion criteria for all subjects were: (i) neuropsychiatric disorders other than MS, (ii) ophthalmological pathologies (i.e., diabetes mellitus or glaucoma), (iii) history of alcohol or drug abuse, (iv) contra-indications to MRI. Twenty-one patients with baseline and one-year clinical and MRI examination were included in the analysis.

### Clinical assessment

All subjects underwent clinical and MRI assessment on the same day. Clinical disability was assessed with the EDSS, 25-foot walk and 9-hole-peg tests (25-FWT and 9-HPT) at baseline, month six and month 12. To confirm sustained disability progression, patients were further assessed during a clinical follow-up visit 12 months after study termination. Clinical worsening was defined as EDSS score increase of one point if the baseline EDSS score was less than or equal to five, or an increase of 0.5 if it was greater than five, or change of >20% for 25-FWT or change of >20% for 9-HPT scores. Disability progression was defined as clinical worsening (i) at month six compared with baseline, confirmed at month 12 or (ii) at clinical follow-up visit 12 months after study termination compared with month 12.

### MRI acquisition and analysis

MRI was performed using a 3.0 T scanner (Philips Achieva, The Netherlands) with an 8-channel SENSE phased-array head coil (Philips Achieva, The Netherlands). The MRI protocol included the following sequences: **a**) axial dual echo TSE sequence: TR = 2500 msec, TE1 = 10 msec, TE2 = 80 msec, FOV = 230x230 mm, matrix size = 512x512, 46 contiguous 3 mm-thick slices; **b**) sagittal 3D T1-weighted turbo field echo sequence: TR = 7.5 msec, TE = 3.5 msec, TI = 900 msec, flip angle = 8°, voxel size = 1x1x1 mm, 172 contiguous slices; **c**) phase-sensitive inversion recovery sequence (PSIR): TR/TE/TI = 4500/8/400 ms, 46 contiguous 3-mm-thick slices with in-plane reconstructed resolution of 0.5x0.5 mm. The same protocol was used for both baseline and follow-up, and patients were repositioned according to established guidelines[14].

Brain T2 and T1 lesion volumes (LV) were measured as previously described[15]. Cerebellar GM and WM lesions were identified respectively on PSIR and dual echo/3D T1 scans by consensus of two examiners (M.P., E.M). All the lesions involving the cortex (either purely intracortical or extending in the WM) were pooled together in the analysis[6]. Cerebellar lesion loads were measured following the same steps applied for whole brain LV quantification.

Normalized brain, GM and WM volumes (NBV, GMV and WMV) were measured on T1-weighted-lesion-filled and non-uniformity corrected images using SIENAX[16] while cerebellar cortical and WM volumes (CCV and CWMV) were measured using Freesurfer (version v5.3.0) longitudinal pipeline[17,18]. Volumes percentage change at follow up was computed, as suggested in Freesurfer, according to the following formula: percentage change = rate of change/volume at baseline, where rate of change = (volume at follow up–volume at baseline)/(time2 –time1).

After Freesurfer analysis, all images were reviewed to maintain accuracy and consistency of volume calculation. The volume-based stream outputs were reviewed and manually edited by two experienced operators according to published guidelines[19,20]. Briefly, the optimal contrast between GM, WM and CSF was obtained by adjusting the contrast intensity window before manual editing. The right and left cerebellar hemispheres were outlined separately. Since it was difficult to distinguish deep cerebellar nuclei from the WM of cerebellar peduncles due to a limited imaging contrast resolution, they were included in the WM segmentation. Image resolution also limited the segmentation of WM peripheral lamellae from cerebellar cortex, since both tissues were often included in the same voxel. When evaluating the postero-lateral borders of cerebellar hemispheres, venous structures that run strictly adjacent to them, such as the transverse and sigmoid sinuses, were followed along each plane in order to exclude them from the final segmentation.

The flocculo-nodular lobule was carefully recognized and entirely included in the cerebellar segmentation.

**Fig 1:**
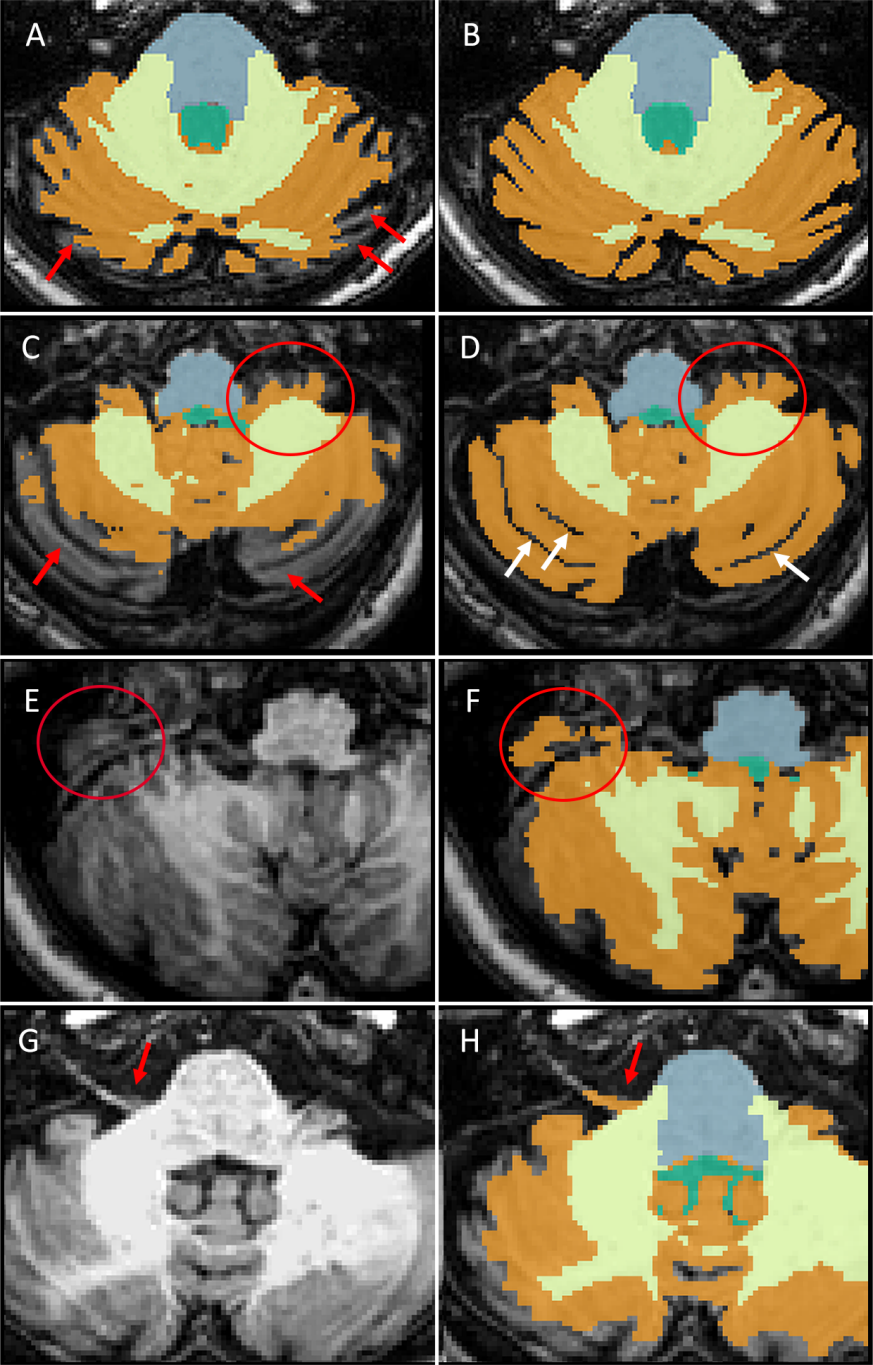
Manual editing of Freesurfer automated segmentation. A-B. Axial T1 of the cerebellum with original (A) and manual edited (B) output volumes of Freesurfer segmentation overlayed. Red arrows show several cerebellar folia not recognized by the automated segmentation. C-D. Axial T1 of the cerebellum with original (C) and manual edited (D) output volumes of Freesurfer segmentation overlayed. Red arrows show several cerebellar folia not recognized by the automated segmentation of Freesurfer. Ellipses show how flocculo-nodular lobule of the cerebellum is not entirely recognized by the automated segmentation. White arrows show some manually segmented sulci. E-F. Axial T1 of the cerebellum without (E) and with (F) the original output volume of Freesurfer segmentation overlayed. Ellipses show how transverse sinus is misrecognized as a part of the cerebellum. Proper manual editing was provided to avoid this issue. G-H. Axial T1 of the cerebellum without (G) and with (H) the original output volume of Freesurfer segmentation overlayed. Red arrows show how VII and VIII cranial nerves are misrecognized as a part of the cerebellum. Proper manual editing was provided to avoid this issue.

If included in the cerebellar automated segmentation, root entry zone of cranial nerves from V to IX, were manually removed. In presence of wide cerebellar sulci, automated segmentation failed in recognition of the posterior part of cerebellum. Proper editing was thus provided and, when needed, sulci were delineated manually in-between cerebellar folia (only when sulci were equal or wider than one pixel namely circa 1mm) (Fig 1).

To assess intra-rater and inter-rater reproducibility and reliability of the cerebellar volume measurements obtained from the manual editing of Freesurfer volume-based stream outputs, cerebellar segmentation with Freesurfer and manual editing was repeated twice with a two weeks interval on the images of five patients by two independent and experienced examiners (M.P. and E.M).

### Statistical analysis

Statistical analysis was performed using SPSS 22.0 (SPSS, Chicago, IL).

Mann-Whitney and Fisher exact test were applied to assess differences in terms of age and gender between patients and CTRLs. An analysis of variance model was applied to investigate differences in MRI metric between patients and controls at baseline, taking into account age and gender as covariates. The cross-sectional between-group comparison of cerebellar volumes was additionally corrected for estimated intracranial volume (ICV), derived from Freesurfer, entering ICV as covariate of no interest in the analysis. Correlations between MRI metrics at baseline and follow-up were tested using non-parametric Spearman test.

Intra-rater and inter-rater reproducibility and reliability of the cerebellar volume measurements obtained from the manual editing of Freesurfer-volume based stream outputs were measured using the coefficient of variation (CV) and the intra-class correlation coefficient (ICC).

Changes in brain and cerebellar volumes over one-year follow-up were assessed using non-parametric Wilcoxon test and the changes were tested for their correlation with disability worsening by a logistic regression. All p values are reported as two-sided significance levels and considered statistically significant when p≤0.05. Sample sizes for given treatment effects and power were calculated using parameters estimated from the sample.

### Standard protocol approvals, registrations, and patient consents

Written informed consent was obtained from all participants before the beginning of the study procedures, according to the Declaration of Helsinki. The protocol was approved by the Institutional Review Board of the Icahn School of Medicine at Mount Sinai.

## Results

No significant group differences were observed when comparing CTRLs and patients with PP-MS with regard to age and sex (p = 0.1). Patients’ clinical features at the two time points are listed in Table 1. Eight out of the 21 patients who repeated MRI at one-year follow up exhibited sustained disability progression based on either EDSS (n = 2), 25-FWT (n = 3), 9-HPT (n = 1) or on both 25-FWT and 9-HPT worsening (n = 2).

**Table 1:** Clinical characteristics of PP-MS patients at baseline and follow-up.

	Baseline (21)	1-year FU (21)
**EDSS median (range)**	4.0 (1.5–6.0)	4.0 (2.0–6.0)
**EDSS cerebellar subsystem median (range)**	1.0 (0.0–3.0)	2.0 (0.0–3.0)
**9-HPT dominant hand, seconds**	30.8±12.2	33.4±17.9
**9-HPT non-dominant hand, seconds**	34.7±17.3	35.9±26.7
**25-FWT, seconds**	7.0±2.1	7.0±2.2

Abbreviations: EDSS = Expanded Disability Status Scale; 9-HPT = 9-Hole Peg Test; 25-FWT = 25-FootWalkingTest. Unless specified, all values are expressed as mean ± SD.

Among the eight patients showing sustained disability at one-year follow up, three were under immunomodulatory treatment.

### MRI measures at baseline and 1-year FU

Compared to CTRLs, NBV, GMV, WMV volumes were lower in PP-MS patients (respectively 1376.88± 69.30 vs 1434.06±53.67 mL, p<0.05; 728.99±43.86 vs 757.31±38.95 mL, p<0.01; 647.88±45.16 vs 676.75±37.02 mL, p>0.05). Likewise, cerebellar volume and CCV were significantly lower (respectively 114.42±12.52 vs 121.93±15.23 and 86.90±8.88 vs 92.77±11.72 mL, p<0.05) while CWMV did not differ between the two groups (27.52±5.26 vs 29.16±4.32 mL, p>0.05). Over one-year, a significant difference was detected in terms of cerebellar T2LV (0.11±0.20 vs 0.13±0.26 mL, p<0.01) and cerebellar T1LV (0.09±0.19 vs 0.11±0.21 mL, p<0.05) but not in terms of PSIR LV (0.06 ± 0.10 vs 0.07 ± 0.10 mL, p = 0.20). Additional patients’ MRI measures at baseline and one-year follow-up are summarized in Table 2.

**Table 2:** Baseline and one-year follow-up MRI findings.

	Baseline	1-year FU	% change	P values*
**NBV, mL**	1376.88±69.30	1372.06±65.21	-0.17±1.60	0.217
**NGMV, mL**	728.99±43.86	727.44±42.36	-0.07±2.17	0.274
**NWMV, mL**	647.88±45.16	644.61±42.76	-0.26±1.86	0.274
**T2LV, mL**	6.00±7.94	6.67±8.61	9.50±25.44	0.056
**T1LV, mL**	3.33±5.24	4.03±6.48	35.23±82.53	0.002
**CL volume, mL**	0.50±0.47	0.48±0.48	0.54±22.19	0.936
**Cerebellar volume, mL**	114.42± 12.52	111.84±12.03	-1.90±1.11	0.0001
**CCV, mL**	86.90±8.88	85.13±8.27	-1.68±1.41	0.0001
**CWMV, mL**	27.52±5.26	26.70±5.15	-2.52±1.95	0.0001

Abbreviations: NBV = normalized brain volume; NGMV = normalized gray matter volume; NWMV = normalized white matter volume; CL = cortical lesion; LV = lesion volume; CCV = cerebellar cortex volume; CWMV = cerebellar white matter volume. All values are expressed as mean ± SD.

*Wilcoxon signed rank test.

The intra-rater CV and ICC for cerebellar volume were 7.78%, 0.95 with an inter-rater ICC of 0.91 (p<0.001).

At follow-up, cerebellar volume was significantly correlated with cerebellar T2LV (r -0.43, p = 0.05), T1LV (r-0.51, p<0.05) but not with PSIR LV (r -0.34, p = 0.13). CCV showed significant correlations with the change of cerebellar T2LV (r -0.53, p<0.01) and T1LV (r -0.50, p<0.05) but not with cerebellar PSIR LV (r -0.17, p = 0.48). CWMV was correlated to cerebellar T2LV (r -0.44, p<0.05), T1LV (r -0.56, p<0.01), and PSIR LV (r -0.48, p<0.05). In addition, cerebellar volume was correlated with brain WMV (r 0.56, p<0.01), but not with NBV, GMV (p>0.1). CWMV was correlated with brain WMV (r 0.58, p<0.01), but not with NBV or GMV (p = 0.1) and CCV did not show any significant correlations with brain volumes (p>0.13 for all).

### Relationship between one-year changes in MRI metrics and clinical outcome

**Fig 2:**
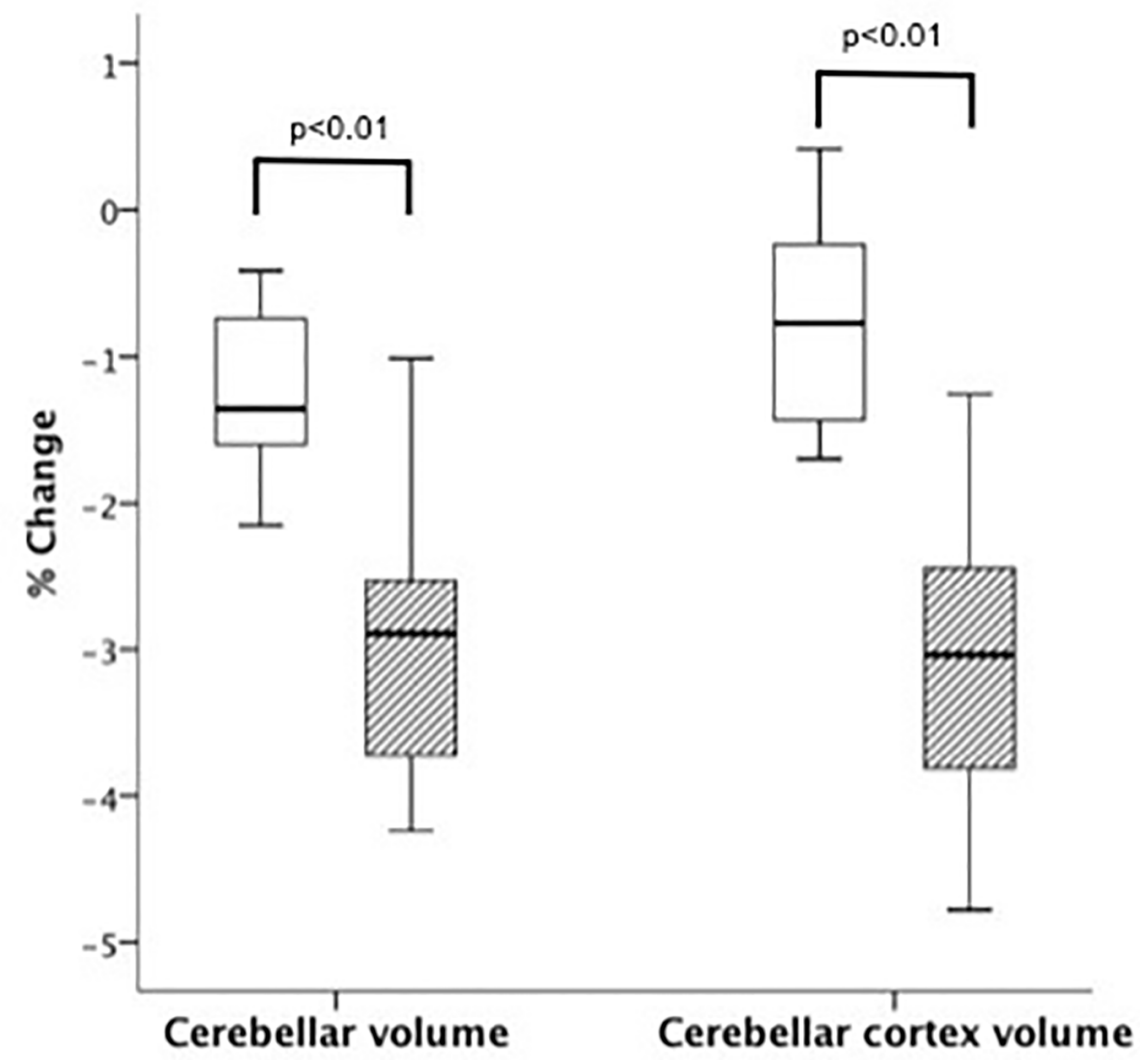
Cerebellar volume and cerebellar cortex volume percentage change for progressed and not- progressed patients. Box plots displaying the 25% to 75% values (boxes) ± 95% values (whiskers), median values (horizontal lines within boxes) of cerebellar volume and cerebellar cortex volume percentage change for progressed (hatched box) and not-progressed (empty box) patients.

Over one-year, significant changes were detected in brain T1LV (p<0.01), cerebellar T2LV (p<0.01) and cerebellar T1LV (p<0.05), cerebellar volume, CCV and CWMV (p<0.001) (Table 2). However, only global cerebellar volume and CCV percentage change were significantly reduced in clinically progressed patients when compared to not-progressed patients (p<0.01; respectively AUC of 0.91 and 0.96) (Fig 2).

### Sample size estimates for cerebellar volumes for clinical trials

Based on the percentage change in cerebellar volume and CCV at one-year follow up we calculated preliminary estimates of sample sizes for clinical trials. The numbers of patients required to detect a 30% effect are 81 per group for cerebellar volume and 162 per group for CCV, assuming 90% power and type 1 error alpha = 0.05 with equal numbers of patients per group.

### Validation PP-MS cohort

We tested the reproducibility of decrease of cerebellar volume in an independent cohort of 20 PP-MS patients from the Sheba Medical Center who presented with clinical characteristics similar to those of our group of PP-MS patients (Table 3). All patients underwent MRI scan and clinical evaluation with the assessment of the EDSS score at baseline and at 1-year follow-up. MRI was performed on a 3.0 T scanner (Signa HDxt-General Electric Milwaukee, WI, USA) with an 8-channel head coil (Signa HDxt- General Electric Milwaukee, WI, USA)] according to the following protocol: a) sagittal 3D T1-weighted fast spoiled gradient echo (FSPGR) sequence: TR = 6.5 msec, TE = 2.1 msec, TI = 450 msec, flip angle = 20°, voxel size = 1x1x1 mm, 255 contiguous slices; b) axial FSE sequence: TR = 4400 msec, TE = 101 msec, FoV = 230x230 mm, matrix size = 256x256, 56 contiguous 2.6 mm-thick slices. At baseline, the cerebellar volume was 133.29±14.05 mL, the CWMV was 31.26±4.28 mL and the CCV was 102.04±12.30. At 1-year follow-up the cerebellar volumes were respectively 131.70±14.58 mL, 31.15±4.17 mL and 100.54±12.59 mL. Over one-year, a significant difference was detected in terms of cerebellar volume (p = 0.009), CCV (p = 0.002), but not CWMV (p = 0.852).

**Table 3:** Demographics and clinical characteristics of patients and controls.

	Exploration cohort	Validation cohort	Controls
**Age, (range)**	50.8±10.98 (32–65)	50.9±11.3 (25–68)	51.1±9.8 (34–63)
**Gender**	9M/12F	9M/11F	9M/11F
**Disease duration,**	9.1±4.9	11.1±7.4	-
**EDSS, median (range)**	4.0 (1.5–6.0)	4.0 (1.5–6.5)	-

Abbreviations: M = male; F = female; EDSS = Expanded Disability Status Scale. All values are expressed as mean ± SD, unless otherwise specified.

The finding of significant changes in cerebellar volume at one-year follow-up in this validation cohort strengthens the reliability of our results. The changes in cerebellar volume were consistent in terms of directionality and magnitude of absolute and percentage change (exploration cohort: cerebellar volume -1.90±1.11%, cerebellar CCV -1.68±1.41%; validation cohort: cerebellar volume -1.47±2.30%, CCV -1.56±2.23%). Although in the validation cohort progressed patients showed higher volumetric percentage change than non-progressed patients for both cerebellar volume (respectively -2.92±3.66% and -1.09±1.85%) and cerebellar CCV (respectively 2.96±3.97% and -1.21±1.69%), considering that only three out of 20 PP-MS patients in the validation cohort exhibited sustained disability progression at one-year follow-up, we did not have the power to replicate the association between changes in cerebellar volume and changes in clinical measures observed in the exploration cohort.

## Discussion

The main findings of our study are the presence of a significant decrease of total and cortical cerebellar volumes over one-year follow-up in patients with PP-MS and the association between the decrease of cerebellar volume and the worsening of clinical measures.

Neuropathological examination of post-mortem brain tissue has revealed that cerebellum is a major predilection site of demyelination in MS, especially in patients with PP-MS and SP-MS[2].

The importance of cerebellar involvement and its contribution to disease-related impairment in MS, especially in progressive forms of the disease has been confirmed by several cross-sectional MRI studies that have demonstrated a significant reduction in cerebellar volume in MS patients when compared with healthy controls[21–23] and a marked reduction in CCV in SP-MS compared with benign MS and CIS[4,24,25].

Despite its relevance, the dynamic of cerebellar volumes over time remains elusive. This is due to the peculiar anatomy of the cerebellum where the tightly folded cortex and its poor demarcation from the white matter challenges the segmentation techniques. However, these challenges have been partly overcome by the better spatial resolution due to the advent of high field MRI, improved gradients, coils, and MRI sequences as well as advancements in data post-processing techniques[26,27]. In our study, we adopted the last released version of the Freesurfer longitudinal pipeline, choosing to apply a widely used brain structure segmentation method. Freesurfer out-streams were visually checked and manually edited by two experienced operators after several hours of training. First, we selected a brief set of guidelines based on the ones provided by de Macedo Rodrigues et al. and Bogovic et al.[19,20] for the manual segmentation of the cerebellum. Then we identified two experienced operators who underwent training of the Freesurfer out-streams manual editing based on the selected guidelines. Finally, we established the intra- and inter-rater reproducibility of the manual editing of the Freesurfer volume-based out-streams. Although we are aware that a correct segmentation of the thin cerebellar gyri and sulci is very difficult and that partial volume voxels may limit the detection of subtle changes in the folia structure, we believe that our results are promising because we found significant changes not only of the CCV but also of the entire cerebellar volume and because we were able to validate our findings relative to the cerebellar changes over time in an independent group of PP-MS patients with similar clinical characteristics. Unfortunately, the small number of patients (n = 3) showing clinical progression in this group, precluded us from testing the association between the decrease of cerebellar volume and the worsening of clinical measures in the validation cohort; however, the higher cerebellar volume loss in the three clinically progressed patients compared to the ones who did not progress is in line with our results in the exploration cohort.

Although almost all PP-MS patients presented some degree of cerebellar involvement at baseline, only five of them experienced a clinical worsening of cerebellar function at follow-up as reflected by the EDSS cerebellar functional system score. In addition, the majority of patients presented focal lesions in cerebellar white matter and/or gray matter. We did find statistically significant associations between cerebellar volumes and local lesion volumes suggesting that the progressive cerebellar atrophy is related to the degeneration of afferent, efferent and association fibers transected in local lesions. Additionally, the association between cerebellar volumes and brain white matter volume suggests that cerebellar atrophy may also result from the degeneration of regions that are remote but connected to the cerebellum through multiple afferent and efferent tracts. This is in line with the findings of a previous study in PP-MS patients showing that reduced fiber coherence, measured by means of DTI tractography, affects the main cerebellar connections (i.e. middle and superior cerebellar peduncles), which mediate important brain functions such as upper limb motor dexterity and speed of walking[22]. Hence, due to the strategic position of the cerebellum in the motor, coordination and cognitive networks, the association that we identified between cerebellar atrophy and sustained disability worsening may be explained not only by the local focal and diffuse WM and GM injury but also by the damage to the multiple connections to the forebrain, the thalamus, and the spinal cord[28–30].

In summary, our results suggest a role for both cerebellar cortex and whole cerebellar volume as a potential short-term imaging measure to monitor treatment effect in clinical trials. Hence, if confirmed in a larger sample of patients, measures of cerebellar volume could provide a short-term outcome for screening experimental treatments in clinical trials of PP-MS with relatively small sample sizes and serve as a potential phase II MRI marker for progressive MS clinical trials.
